# Hereditary multiple exostoses and solitary osteochondroma associated with growth hormone deficiency: to treat or not to treat?

**DOI:** 10.1186/s13052-015-0162-2

**Published:** 2015-08-04

**Authors:** Mauro Bozzola, Chiara Gertosio, Maria Gnoli, Federico Baronio, Elena Pedrini, Cristina Meazza, Luca Sangiorgi

**Affiliations:** Internal Medicine and Therapeutics Department, Pediatric and Adolescent Unit, University of Pavia, Fondazione IRCCS Policlinico San Matteo, Piazzale Golgi, 2, 27100 Pavia, Italy; University of Pavia, Fondazione IRCCS Policlinico San Matteo, Pavia, Italy; Pediatric Endocrinology Program, Pediatric Unit, S.Orsola-Malpighi Hospital, University of Bologna, Bologna, Italy; Medical Genetic Department, Rizzoli Orthopaedic Institute [IOR], Bologna, Italy

## Abstract

**Background:**

Osteochondroma generally occurs as a single lesion and it is not a heritable disease. When two or more osteochondroma are present, this condition represents a genetic disorder named hereditary multiple exostoses (HME). Growth hormone deficiency (GHD) has rarely been found in HME patients and a few data about growth therapy (GH) therapy effects in development/growth of solitary or multiple exostoses have been reported.

**Case presentation:**

We describe the clinical features of 2 patients (one with osteochondroma and one with HME) evaluated before and after GH therapy. In the first patient, the single osteochondroma was noticed after the start of treatment; the other patient showed no evidence of significant increase in size or number of lesions related to GH therapy.

**Conclusion:**

It is necessary to investigate GH secretion in patients with osteochondroma or HME and short stature because they could benefit from GH replacement therapy. Moreover, careful clinical and imaging follow-up of exostoses is mandatory.

## Background

Solitary osteochondroma occurs as a sporadic event and it is known to be a non-genetic condition. Osteochondromas account for about 30 % of benign bone tumours and they grow slowly in skeletally immature individuals, while the increase in size of this lesion in adult patients is considered an indication of malignant transformation [1].

Hereditary multiple exostoses (HME) is an autosomal dominant heritable disorder, characterized by the formation of multiple osteochondromas: these lesions can increase in number and size during skeletal development [2] and stop growing with skeletal maturity, after which no new osteochondromas develop. Exostoses are rarely evident at birth [3] and the age of onset is variable, from 2 to 15 years [4]. Clinical expression of HME phenotype is variable in individuals, also in the same family [5, 6]. The majority of HME or solitary osteochondroma patients are asymptomatic. Osteochondromas are located in bones that develop from cartilage, especially the long bones of the extremities, predominantly around the knee. Osteochondromas can cause deformities, bursa formation, arthritis and impingement on adjacent tendons, nerves, vessels [3–5, 7]. However, the most serious complication is the malignant transformation towards a rare form of bone cancer called chondrosarcoma, described in adulthood with variable incidence in different series, but still in a small percentage in HME patients (0.5-5 %) [5, 8]. Solitary osteochondroma malignant transformation is estimated in about 1-2 % [9].

HME is caused by mutations in two genes, *EXT1* (MIM *608177) and EXT2 (MIM *608210) [5, 10, 11]; however, in some families no mutations in *EXT1* and *EXT2* genes are detected, suggesting involvement of other genes in the pathogenesis of the disease. While most patients have an affected parent, in other cases patients carry a *de novo* mutation. *EXT1* and *EXT2* proteins are ubiquitously expressed transmembrane proteins, which form heterodimers. These proteins are involved in heparan sulfate (HS) elongation and related to signal transduction cascade for regulation of chondrocyte differentiation, ossification, and apoptosis [12]. On the contrary, solitary osteochondroma somatic mutations in the *EXT1* gene, are extremely rare [10].

In comparison with the reference healthy population and patients’ parents or sibs, a shorter stature has been reported in some HME patients [13], but in other studies most adults with *EXT2* mutations and many with *EXT1* mutations fall within the normal range [14]. Pedrini and colleagues described a correlation between reduced height and clinical severity of disease, and patients with no detectable *EXT*1 or *EXT2* mutation had a nearly normal height in this series [8]. Further studies are needed to confirm the incidence of short stature in HME patients.

In the present study, we collected data about the clinical follow-up of HME and solitary osteochondroma patients with growth hormone deficiency (GHD) who underwent GH therapy; in particular, we investigated their response to therapy and the effects on osteochondroma development and growth, observing the bone lesions before and during GH treatment. Furthermore, the question whether the occurrence and/or increase of the exostoses may be related to GH treatment or to the normal evolution of this bone disease is debated.

## Case presentation

We followed two GHD children (one boy and one girl). GHD was established, after excluding other causes of growth failure, on the basis of short stature, reduced growth rate, delayed bone age and insufficient GH response to at least two classic pharmacological stimuli (peak <10 ng/ml), according to international guidelines in force at that time [15]. Recombinant hGH was administered by daily subcutaneous injections, in according to national rules for the use of GH [16]. GH replacement treatment was monitored every six months by evaluating anthropometric and laboratory parameters (i.e. insulin-like growth factor-I (IGF-I) levels, A1c fraction of glycosylated haemoglobin, adrenal and thyroid function), and assessing compliance.

**Fig. 1:**
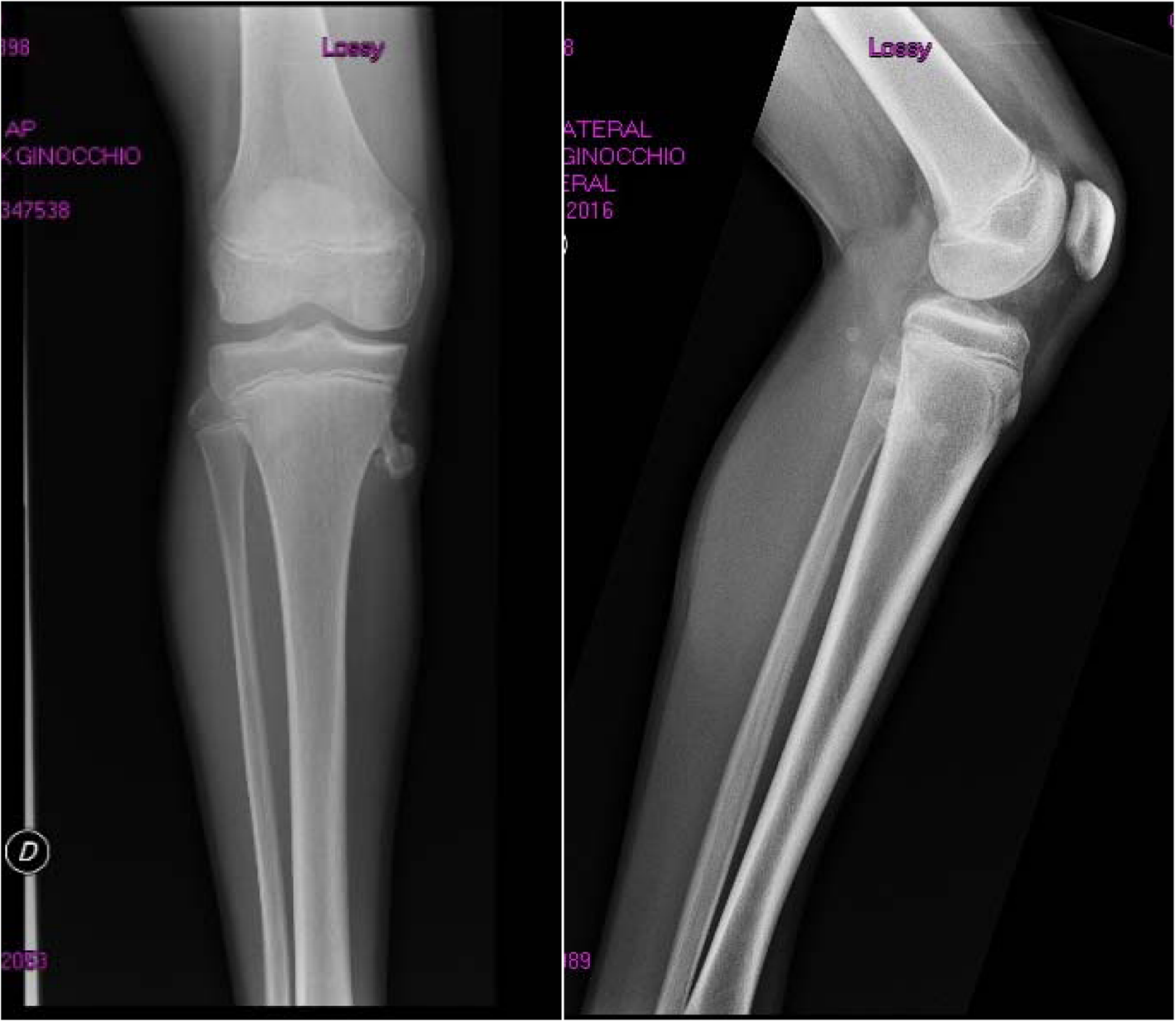
Patient 1 right leg X-rays during GH treatment

**Fig. 2:**
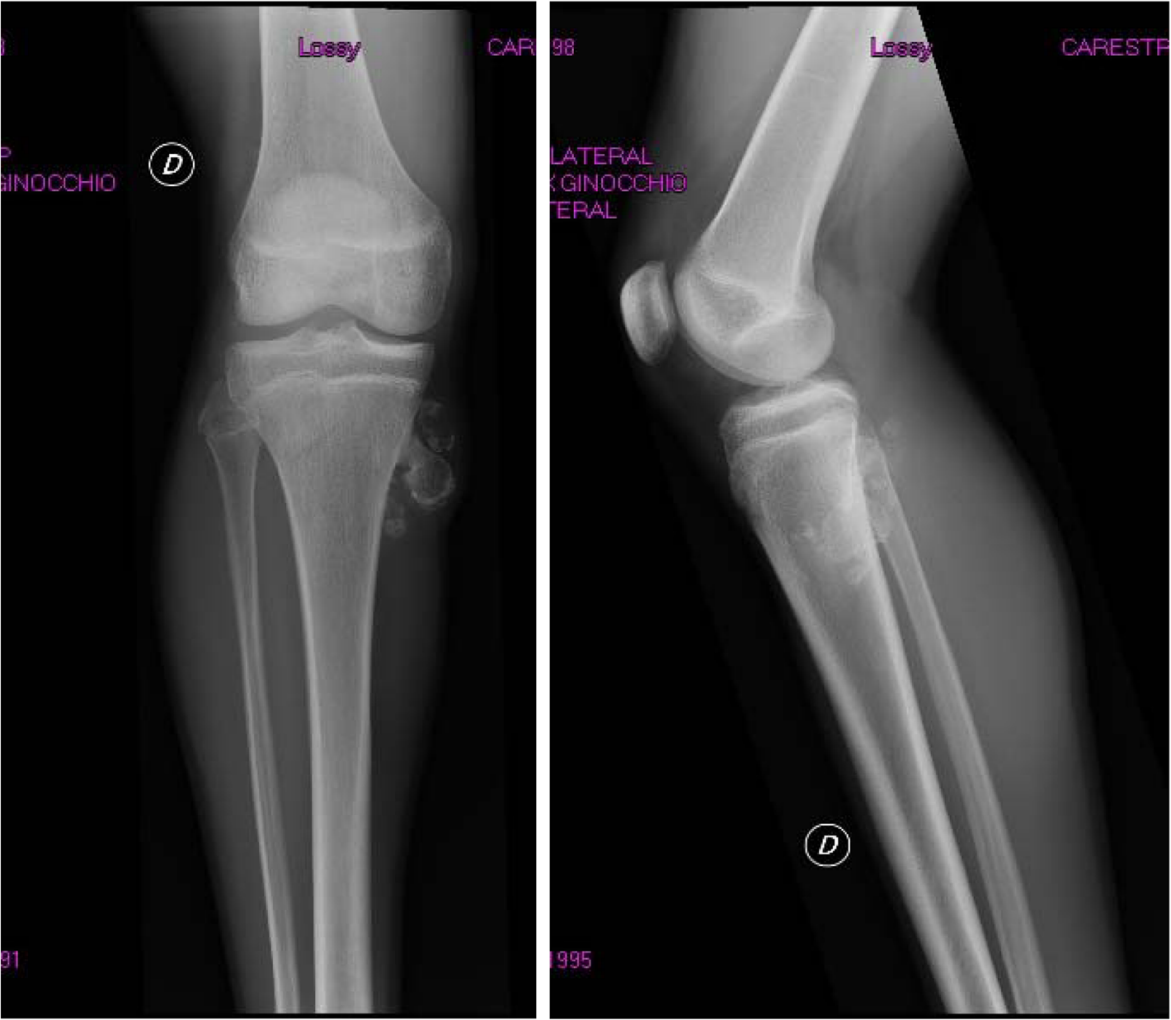
Patient 1 right leg X-rays after GH treatment

Patient 1 was born by spontaneous vaginal delivery at the 38^th^ week of an uncomplicated pregnancy and he was the fourth child of non-consanguineous parents. Birth length was 52 cm and birth weight 3,200 g. Perinatal period and neuropsychological development were normal. Scholastic performance and physical activity were normal, too. The patient was first referred to the Paediatric department at the age of 13 years and 9 months because of slight short stature (150 cm, -1.11 SDS). He showed a weight of 34 Kg (BMI 15.11 kg/m^2^, -2.0 SDS), a reduced growth rate (4 cm/year; -4.0 SDS), a delayed bone age (11 years) and gonadal volume of 3.5 ml (pre-pubertal stage). Genetic target was 181.75 cm (1.1 SDS). Hypothyroidism, chronic diseases, malabsorption including celiac disease and skeletal dwarfism were excluded. GH deficiency was diagnosed by two classic stimulation tests such as arginine infusion (GH peak: 6.8 ng/ml) and glucagon administration (GH peak: 6.1 ng/ml) and confirmed by low serum IGF-I values (132 ng/ml; -3.19 SDS). The MRI of the hypothalamic-pituitary region showed major abnormalities including reduced size of the pituitary gland and the thickening of the pituitary stalk, but normal appearance of neurohypophysis. A replacement therapy with GH (0.21 mg/Kg/week divided in 6 doses) showed a good response on linear growth: 6.58 cm (1.23 SDS) during the first year and 6.80 cm (5.08 SDS) during the second year of therapy. At the age of 15 years and 7 months, i.e. 1.5 years after the beginning of GH treatment, a cartilage-capped pedunculated solitary osteochondroma (12 mm) on the medial margin of the third proximal of the right tibial diaphysis in communication with two small solid palpable cartilaginous formations (15×18 mm and 8×5 mm) was observed (Fig. 1). The orthopaedist decided to immediately stop GH therapy because he was afraid of any influence on the exostoses. Since no macroscopic increase of the exostoses was observed after 4 months, the patient’s parents requested that the replacement therapy be started again. The patient grew 7.4 cm more and decided to stop GH therapy at the age of 18 years when he reached a height of 177.4 cm [0.41 SDS], although skeletal maturity was not achieved. After stopping the treatment, right leg X-rays showed an increased size of the pedunculated exostoses on the medial margin of the third proximal of the right tibial diaphysis (31×20.5 mm) (Fig. 2) compared to previous radiographs performed during the treatment (20.2×16.6 mm) in communication with other palpable cartilaginous formations (11.6×8.2 mm, 8.5×7 mm, 17×10 mm) of increased size compared to previous radiographs.

The family history in this case is peculiar, because other family members had solitary osteochondromas. The proband’s older brother (23 years old) had a solitary osteochondroma on the same side as the proband. Bilateral X-rays of knee, ankle and hand did not show further exostoses and no other lesions have been reported. Exostoses was revealed at the age of 13 years and slightly increased 5 years later. X-rays in both parents performed for traumatic events did not show osteochondromas. It was reported that both the paternal grandmother and the maternal grandfather had the same solitary osteochondroma as the proband’s brother (no clinical reports available). The 24-years-old sister showed a bone protuberance on the sole of the right foot, painful during exercise, while the younger sister, aged 21, showed no visible exostoses. A first cousin of the proband underwent treatment for a brief period for GH deficiency, but at the age of 19 years her height was reported to be about 160 cm (-0.59 SDS). Because of the unusual recurrence of the lesion in other family members, *EXT1* and *EXT2* molecular analysis was performed in the proband: no sequence variants or deletion were detected.

Patient 2 was a girl affected by HME who came to our attention when she was 3 years and 3 months old. Height was 95 cm (-0.05 SDS), weight 12.5 Kg (BMI 13.85 kg/m^2^, -1.38 SDS). She had small osteochondromas (scapulae, ribs, left distal femur, right proximal tibia, left forearm) at clinical examination. Family history was positive for the disease: the father and the younger sister were also affected. She was born by spontaneous vaginal delivery at term of an uncomplicated pregnancy. Birth weight was 2,880 g. Perinatal period and neuropsychological development were normal. Genetic target was 158.5 (-0.7 SDS). The child underwent regular orthopaedic follow-up in particular to evaluate the larger forearm exostoses for surgical removal. Furthermore, she had diagnosis of Langerhans cell histiocytosis at the age of 3 years and 6 months. She also had a follow-up and therapy for diabetes insipidus (Minirin 0.075 mg twice day). At the age of 5 years and 5 months auxologic parameters were: height 104 cm (-1.22 SDS) and weight 17 Kg (BMI 15.72 kg/m^2^, -0.09 SDS). Therefore an endocrinological evaluation was started. Clonidine test showed a GH peak of 3.6 ng/ml, arginine test results showed a GH peak of 1.7 ng/ml; IGF-I levels were 66 ng/ml (-1.62 SDS). The MRI of the hypothalamic-pituitary region showed the thickening of the pituitary stalk (4.18 mm) and normal appearance of anterior pituitary, with the absence of the neurohypophysis bright spot. At age of 6 years she started GH replacement treatment at a standard dose of 0.24 mg/kg/week. Before starting GH therapy an ultrasound examination of the osteochondromas was performed. After the first year of treatment growth velocity was 9.5 cm/year (5.9 SDS) and her height was 118 cm (-0.72 SDS). During the second and third year of GH treatment growth velocity decreased to 1.8 and 2.1 SDS, respectively. At 6, 12 and 18 months after the treatment, ultrasound evaluation showed no major modifications of exostoses, but only a mild thickening of the left ulnar and humeral exostoses cartilaginous cap after 18 months. Molecular analysis identified *EXT1* mutation (c.1019G > A; p.R340H), previously found in her father.

## Discussion and conclusions

Osteochondromas are one of the most common benign bone tumours slowly growing in skeletally immature individuals. In adult patients, the increase in size of an osteochondroma is considered an indication of malignant transformation. On the other hand, spontaneous disappearance of exostoses in childhood or puberty has been reported [1, 17], but during skeletal development the appearance of new lesions or increased size are a part of the natural history of HME. In a large series of patients, a few genotype-phenotype correlations have been described [8]. Actually, HME is a disease with wide inter- and intra-familial clinical variability, so age of onset, number, size and localization of osteochondromas cannot be predicted on molecular bases.

Because of growth plate involvement, a correlation between growth defect and HME or solitary osteochondroma has been suggested. The growth plate is responsible for longitudinal bone growth and chondrocytes play a role in bone elongation. Chondrocytes function in growth plate and bone growth is tightly regulated by circulating molecules (i.e. GH) and locally secreted factors (in part by chondrocytes) and extracellular matrix (ECM) components (including HS/proteoglycans) are part of the intricate growth plate biology [18]. It has been suggested that osteochondroma development is due to abnormal HS-dependent signalling pathways and related to increased environment factors inducing proliferation in growth plate [19]. The molecular bases of many chondrodysplasias, chondrocytes and ECM components detailed role in all involved signalling pathways in growth plate are not completely understood and further studies are in progress [20]. Because of the GH effect on growth plate biology and chondrocyte proliferation, it could be possible that GH could stimulate osteochondroma development, but no strong evidence has been reported from large patient series.

Whereas short stature has been reported in several studies involving HME patients [6, 13], GH deficiency and GH replacement therapy have not been well described in this disease. Lazaro Martinez reported a patient affected by HME and short stature due to GH deficiency who was treated with GH. Owing to the occurrence of an inappropriate growth of the upper extremities and a worsening of the cervical exostoses without pain and functional impairment, GH therapy was interrupted after 1 year [21]. Galasso and colleagues reported a patient with HME and partial GH deficiency treated for four years. GH replacement therapy was discontinued because of a poor linear growth response and a moderate increase in size of the exostoses. However, the diagnosis of GH deficiency was not confirmed by a third test [22]. Both the authors concluded that a careful follow-up in these patients is mandatory, even if no evidence of adverse effects on disease course was proven in their two patients.

In our patient 1, it was difficult to establish whether the increase of the osteochondroma was due to GH therapy or to the normal evolution of this bone disease. However, the increased size of the exostoses in our GH-treated patient and his untreated brother suggests that age, especially puberty, could be the major factor affecting the evolution of these curious bone lesions. Furthermore, this patient had a familial history of osteochondroma but no alterations have been identified in the EXT1 and EXT2 genes. We could hypothesize that the familial recurrence of osteochondroma could be related to an altered and still unknown genetic background.

In the HME patient (patient 2), no evident increase in size or number of lesions was observed. Moreover, since these lesions tend to increase with age, we are not able to confirm or not if GH treatment could modify the natural course of the disease, inducing growth of existent lesions or new lesion development, since the patient is now at 18 months of treatment.

These data are not conclusive about the effects of GH therapy on the lesions, but they can be preliminary for a larger observational study to define whether this treatment worsens the natural course of the disease or not. Since to the best of our knowledge there is no evidence of either increased growth or development of osteochondroma due to GH therapy and evidence of negative effects of the therapy in both patients with osteochondroma and HME, a closer follow-up is mandatory during GH replacement therapy, in particular in those with osetochondroma since the most serious complication of osteochondromas is the malignant transformation towards chondrosarcoma [5]. In fact, it has recently been found a 5 times more bone tumour-related deaths than expected in adults treated with GH during childhood [23]. Such an effect is biologically plausible because these tumours mostly occur during phases of rapid bone growth, seem to be related to height, and are related to the IGF-I system [24, 25].

In conclusion, it is necessary to investigate GH secretion in both patients with solitary osteochondroma and HME and short stature, because when GH deficiency is confirmed by clinical features and classic pharmacological tests, GH replacement therapy could be started to improve their stature. Moreover, a close follow-up of exostoses and HME before and during a long-term GH treatment is mandatory, in particular in patients with osteochondorma which could transformate into chondrosarcoma. However, studies with a longer follow-up are needed in order to define long-term effects of GH treatment on osteochondromas.

## Consent

Written informed consent was obtained from the parents of the patients for publication of this Case report and any accompanying images.

## Abbreviations

HME: Hereditary multiple exostoses
GHD: Growth hormone deficiency
GH: Growth hormone
HS: Heparan sulfate
IGF-I: Insulin-like growth factor-I
BMI: Body mass index
SDS: Standard deviation score
ECM: Extracellular matrix
